# Supplementary material for: Positive Impacts of Aphanizomenon Flos Aquae Extract on Obesity-Related Dysmetabolism in Mice with Diet-Induced Obesity
Source: Cells. 2023 Nov 25;12(23):2706. doi: 10.3390/cells12232706 (PMC10705513; doi:10.3390/cells12232706)
Supplement: Supplementary file 1 [file cells-12-02706-s001.zip › cells-2619038-supplementary.pdf]

Supplemental Table S1. *Composition of AFA extract.*

**Ingredients (g/Kg)**	
Carbohydrate, %	12,07
Protein, %	36,81
Fat, %	3,73
Fiber, %	3,84
Total Energy, Kcal/100g	229,1
**Ingredients (mg/100 g)**	
Folic Acid	3,73
Thiamine	24
Cyanocobalamin	0,08
Riboflavin	3,52
Piridoxine	2,74
Nicotinic acid	0,37
Vit. K1 phylloquinone	4,81
Calcium pantothenate	0,16
Iodine (μg/g)	18,2
Carotenoids	940
Phenolic compounds	76,1
Isoleucine	824
Leucine	1452
Threonine	911
Lysine	970
Phenylalanine	706
Valine	893
Arginine	1063
Histidine	261
Alanine	1311
Glycine	822
Aspartic acid	201
Proline	806
Serine	820
Glutamic acid	110

Tyrosine	480
Tryptophan	205
AFA-phycocyanins	870
